# Supplementary material for: Long-term relative survival in uveal melanoma: a systematic review and meta-analysis
Source: Commun Med (Lond). 2022 Mar 1;2:18. doi: 10.1038/s43856-022-00082-y (PMC9053233; doi:10.1038/s43856-022-00082-y)
Supplement: Supplementary file 1 — Supplementary Information [file 43856_2022_82_MOESM1_ESM.pdf]

Supplementary tables to

Long-term relative survival in uveal melanoma: a systematic review and meta-analysis

Gustav Stålhammar M.D. Ph.D.[1,2] Christina Herrspiegel M.D.[1,2]

[1]St. Erik Eye Hospital, Box 4078, 171 04 Solna, Stockholm, Sweden,

[2]Department of Clinical Neuroscience, Karolinska Institutet, Tomtebodavägen 18A, plan 5, 171 77 Stockholm, Sweden.

Table of contents:

Page 2……………………………….Supplementary table 1. Estimations of relative survival in a Finnish cohort published by Kujala *et al.*
Page 3………………………………. Supplementary table 2. Data collection methods for included studies.
Page 4………………………………. Supplementary table 3. Combined estimate of relative survival rates 1 through 30 years after uveal melanoma diagnosis.
Page 5………………………………. Supplementary table 4. Estimates of variance of relative survival in included studies.

Year after diagnosis	All-cause mortality	Melanoma-related mortality	Expected survival Swedish reference population	Relative survival	95 % CI upper of observed survival rate	95 % CI lower of observed survival rate	Standard error of observed survival rate	95 % CI upper of relative survival rate	95 % CI lower of relative survival rate	1 minus relative survival
5	0.62	0.69	0.98	0.63	0.68	0.56	0.03	0.66	0.60	0.37
10	0.47	0.60	0.82	0.57	0.52	0.41	0.03	0.61	0.54	0.43
15	0.35	0.55	0.65	0.54	0.40	0.29	0.03	0.58	0.50	0.46
20	0.25	0.52	0.50	0.50	0.30	0.20	0.03	0.55	0.45	0.50
25	0.21	0.51	0.34	0.62	0.26	0.16	0.03	0.69	0.54	0.38
30	0.18	0.50	0.22	0.82	0.22	0.13	0.02	0.92	0.71	0.18

Supplementary table 1. Estimations of relative survival in the Finnish cohort published by Kujala *et al*. (Kujala E, Mäkitie T, Kivelä T. Very long-term prognosis of patients with malignant uveal melanoma. Invest Ophthalmol Vis Sci. 2003;44(11):4651-9.)

#	Data collection method	*n* (%)
1	Population-based registries outside the authors' institution, including cancer registries and cause of death registries	4 (44)
2	Institution's clinical records	0 (0)
3	Combination of 1 and 2	5 (55)
4	Other	0 (0)

Supplementary table 2. Data collection methods for included studies.

Year after diagnosis	Relative survival, %
**1**	95
**2**	90
**3**	86
**4**	82
**5**	79
**6**	76
**7**	73
**8**	70
**9**	68
**10**	66
**11**	64
**12**	63
**13**	62
**14**	61
**15**	60
**16**	60
**17**	59
**18**	59
**19**	59
**20**	60
**21**	60
**22**	60
**23**	61
**24**	62
**25**	62
**26**	63
**27**	64
**28**	65
**29**	66
**30**	67

Supplementary table 3. Combined estimate of relative survival rates 1 through 30 years after uveal melanoma diagnosis.

Variance statistic	5 years	10 years	15 years	20 years	25 years	30 years
Tau^2	0.01	0.01	0.01	0.01	0.01	0.02
Chi^2	241	72	39	13	4	14
I^2	97 %	94 %	92 %	85 %	48 %	86 %

Supplementary table 4. Estimates of variance of relative survival in included studies.
