# Supplementary material for: Long-term relative survival in uveal melanoma: a systematic review and meta-analysis
Source: Commun Med (Lond). 2022 Mar 1;2:18. doi: 10.1038/s43856-022-00082-y (PMC9053233; doi:10.1038/s43856-022-00082-y)
Supplement: Supplementary file 5 — Description of Additional Supplementary Files [file 43856_2022_82_MOESM5_ESM.pdf]

## Description of Additional Supplementary Files

**File Name:** Supplementary Data 1

**Description**: Source data used to generate figure 2

**File Name:** Supplementary Data 2

**Description**: Source data used to generate figure 3
