# Supplementary material for: Long-term relative survival in uveal melanoma: a systematic review and meta-analysis
Source: Commun Med (Lond). 2022 Mar 1;2:18. doi: 10.1038/s43856-022-00082-y (PMC9053233; doi:10.1038/s43856-022-00082-y)
Supplement: Supplementary file 6 — Reporting Summary [file 43856_2022_82_MOESM6_ESM.pdf]

nature portfolio

Corresponding author(s): Gustav Stålhammar
St. Erik Eye Hospital

Last updated by author(s): January 18, 2022

Reporting Summary

Nature Portfolio wishes to improve the reproducibility of the work that we publish. This form provides structure for consistency and transparency in reporting. For further information on Nature Portfolio policies, see our Editorial Policies and the Editorial Policy Checklist.

Statistics

For all statistical analyses, confirm that the following items are present in the figure legend, table legend, main text, or Methods section.

n/a	Confirmed	
☐	☒	The exact sample size (*n*) for each experimental group/condition, given as a discrete number and unit of measurement
☐	☒	A statement on whether measurements were taken from distinct samples or whether the same sample was measured repeatedly
☐	☒	The statistical test(s) used AND whether they are one- or two-sided<br>*Only common tests should be described solely by name; describe more complex techniques in the Methods section.*
☐	☒	A description of all covariates tested
☐	☒	A description of any assumptions or corrections, such as tests of normality and adjustment for multiple comparisons
☐	☒	A full description of the statistical parameters including central tendency (e.g. means) or other basic estimates (e.g. regression coefficient) AND variation (e.g. standard deviation) or associated estimates of uncertainty (e.g. confidence intervals)
☐	☒	For null hypothesis testing, the test statistic (e.g. *F*, *t*, *r*) with confidence intervals, effect sizes, degrees of freedom and *P* value noted<br>*Give P values as exact values whenever suitable.*
☒	☐	For Bayesian analysis, information on the choice of priors and Markov chain Monte Carlo settings
☒	☐	For hierarchical and complex designs, identification of the appropriate level for tests and full reporting of outcomes
☒	☐	Estimates of effect sizes (e.g. Cohen's *d*, Pearson's *r*), indicating how they were calculated

Our web collection on statistics for biologists contains articles on many of the points above.

Software and code

Policy information about availability of computer code

Data collection	No software was used for data collection.
Data analysis	The statistical analysis was performed using the Review Manager by the Cochrane Collaboration (RevMan Version 5.4. Copenhagen: The Nordic Cochrane Centre; The Cochrane Collaboration, 2014) and SPSS statistics version 26 (IBM, Armonk, NY, USA).

For manuscripts utilizing custom algorithms or software that are central to the research but not yet described in published literature, software must be made available to editors and reviewers. We strongly encourage code deposition in a community repository (e.g. GitHub). See the Nature Portfolio guidelines for submitting code & software for further information.

Data

Policy information about availability of data

All manuscripts must include a data availability statement. This statement should provide the following information, where applicable:

- Accession codes, unique identifiers, or web links for publicly available datasets
- A description of any restrictions on data availability
- For clinical datasets or third party data, please ensure that the statement adheres to our policy

All data used in this review and meta-analysis is available from publicly available and herein referenced sources. All data generated or analyzed during this study are included in this published article and its supplementary information files. Source data used to generate figure 2 and 3 is provided as supplementary Data file 1 and 2, respectively.

Field-specific reporting

Please select the one below that is the best fit for your research. If you are not sure, read the appropriate sections before making your selection.

☒ Life sciences ☐ Behavioural & social sciences ☐ Ecological, evolutionary & environmental sciences

For a reference copy of the document with all sections, see nature.com/documents/nr-reporting-summary-flat.pdf

Life sciences study design

All studies must disclose on these points even when the disclosure is negative.

Sample size	The sample size of of 18 495 patients was arrived at after a systematic review and application of inclusion and exclusion criteria, detailed below.
Data exclusions	Publications were included for full-text screening if they reported 1) ≥ five-year relative survival rates (or data that could be readily converted to relative survival) for patients with uveal melanoma, 2) consecutively or prospectively included patients, 3) at least 100 patients. Studies were excluded if they 1) only reported survival rates for prognostically relevant subgroups (e.g., tumors with specific mutations, gene expression profiles, histological appearance or size categories), 2) were earlier versions of a series of articles from the same database or center, 3) reported patients that were already included in another publication, or 4) did not provide confidence intervals or standard errors for their relative survival estimates. Studies including patients with primary conjunctival or orbital melanomas or metastatic lesions were not considered.
Replication	Attempts at replication were not performed. The variance of survival rates across the population of studies was evaluated with τ2, which reflects the amount of true heterogeneity regardless of number of included studies or sample size. The alternative measurements of heterogeneity Q and I2 were included for comparison.
Randomization	This is a systematic review and meta-analysis. Patients were not allocated into experimental groups.
Blinding	This is a systematic review and meta-analysis. Patients or investigators were not blinded.

Reporting for specific materials, systems and methods

We require information from authors about some types of materials, experimental systems and methods used in many studies. Here, indicate whether each material, system or method listed is relevant to your study. If you are not sure if a list item applies to your research, read the appropriate section before selecting a response.

Materials & experimental systems

n/a	Involved in the study
☒	☐ Antibodies
☒	☐ Eukaryotic cell lines
☒	☐ Palaeontology and archaeology
☒	☐ Animals and other organisms
☒	☐ Human research participants
☐	☒ Clinical data
☒	☐ Dual use research of concern

Methods

n/a	Involved in the study
☒	☐ ChIP-seq
☒	☐ Flow cytometry
☒	☐ MRI-based neuroimaging

Clinical data

Policy information about clinical studies

All manuscripts should comply with the ICMJE guidelines for publication of clinical research and a completed CONSORT checklist must be included with all submissions.

Clinical trial registration	This is not a clinical trial.
Study protocol	The protocol was registered and published in advance on PROSPERO (CRD42021265504).
Data collection	We did a meta-analysis to evaluate the long-term relative survival in uveal melanoma. Data was acquired with a comprehensive literature search in the PubMed, Web of Science and Embase databases for peer reviewed published articles that described relevant results. The following search terms were used and matched to appropriate medical subject headings: ("uveal melanoma" OR "choroidal melanoma" OR "ciliary body melanoma") AND "relative survival". The search strategy was restricted to titles and/or abstracts of human clinical studies published after January 1st 1980 in English or any language for which an English translation was readily available.22 The latest search was performed on August 19, 2021. All available studies were included and could be accessed in full via the University Library, Karolinska Institutet. Study authors were contacted if discrepancies existed, for clarifications or if we thought that additional unpublished data could be useful for this analysis. Trial registries, unpublished studies, grey literature, animal

	studies, laboratory studies, letters to the editor, correspondence, notes, editorials, and conference abstracts were not considered. Reference lists of included articles were searched for additional studies.
Outcomes	The a priori determined outcome measure was the long-term relative survival rate, reported in five-year intervals.
